# Photon‐to‐Heat Energy Harvesting Fluorescent Protein Coatings for Thermoelectrics

**DOI:** 10.1002/advs.202522088

**Published:** 2025-12-08

**Authors:** Anna Zieleniewska, Sriram Kunchapudi, Stephanie Willeit, Rubén D. Costa

**Affiliations:** ^1^ Technical University of Munich Campus Straubing for Biotechnology and Sustainability Chair of Biogenic Functional Materials Schulgasse, 22 94315 Straubing Germany

**Keywords:** biogenic materials, energy harvesting, fluorescent proteins, photothermal conversion, protein‐polymer coatings

## Abstract

Energy‐related protein optoelectronics is an emerging field with interesting examples in photon manipulation in lighting devices, photon‐induced electron transfer in photovoltaics, and H‐transfer in moisture electrical generators. Here, this field is extended with the concept of photon‐to‐heat energy harvesting fluorescent protein (FP) coatings applied to thermoelectric generators (TEG). This device consists of a commercial TEG coated with a FP‐polymer coating optimized in terms of *i*) the amount of a high‐absorbing and low‐emissive FP named E2‐Crimson and *ii*) the thermal features of a mixture of the branched/linear polyethylene oxide matrix. With photothermal efficiencies of 48% the FP‐coatings can reach temperatures between 40–60 °C under monochromatic (590 nm) and the white LED illumination sources. This results in a total power gain of up to 232 µW cm^−2^ (white LED), highlighting the potential of FP‐coatings in sustainable energy harvesting for low‐power autonomous devices. Overall, this work provides the first stepping stone in protein‐based photon‐to‐heat conversion materials, which promise easy‐to‐tune photothermal properties through the choice of the protein chromophore, the engineering of the protein scaffold, and the control of the protein‐polymer interactions.

## Graphical abstract

Fluorescent proteins (FPs) are used in light‐absorbing coatings on thermoelectric generators (TEGs) to convert light into heat and generate electricity. By tuning the properties of FP‐doped polymer matrices, photothermal efficiencies of up to 48% have been achieved. This biohybrid strategy offers a sustainable approach for powering low‐energy devices using customizable protein‐based photon‐to‐heat conversion materials.

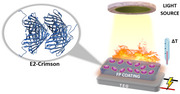

## 1 Introduction

In our interconnected world, technology plays a pivotal role in every aspect, significantly influencing our growing energy needs and overall Earth's wealth.^[^ ^1^ ^]^ Thus, adopting renewable energy sources with enhanced efficiency is mandatory.^[^ ^2^ ^]^ In this context, energy harvesting materials offer environmentally friendly solutions for decentralized power generation by capturing underutilized energy resources, including ambient light,^[^ ^3^ ^]^ thermal variations,^[^ ^4^ ^]^ moisture fluctuations,^[^ ^5^ ^]^ and kinetic energy from everyday movements.^[^ ^6^ ^]^ They are especially valuable for powering small devices and sensors in remote or hard‐to‐reach locations. Indeed, they could become a key source of power for the rapidly expanding of the Internet of Things (IoT).^[^ ^7^ ^]^

Among these strategies, light‐to‐electricity conversion is one of the most widely developed, proceeding via two primary routes: photovoltaics (PVs) and photothermal energy conversion. On one hand, indoor PVs have achieved conversion efficiencies exceeding 35% under low‐light conditions, making them well‐suited for powering devices such as sensors and communication nodes.^[^ ^7^, ^8^, ^9^, ^10^ ^]^ On the other hand, photothermal coatings absorb light and convert it into heat. This heat can then be used to generate electricity when coupled to a thermoelectric generator (TEG), offering sustainable alternatives for indoor energy harvesting applications.^[^ ^11^, ^12^, ^13^, ^14^, ^15^, ^16^, ^17^, ^18^, ^19^ ^]^ This approach offers several distinct advantages, particularly under conditions where light intensity is diffuse or intermittent, or where heat management is a desirable byproduct.

Photothermal materials generally fall into two broad categories, each defined by their target applications and associated trade‐offs. The first category includes high‐performance systems developed primarily for biomedical applications. Examples include gold nanorods, which are used in photothermal therapy due to their strong localized surface plasmon resonance;^[^ ^20^ ^]^ semiconducting polymers like polypyrrole and polyaniline;^[^ ^21^ ^]^ and dye‐loaded nanoparticles based on indocyanine green or croconaine dyes.^[^ ^22^, ^23^ ^]^ These materials achieve photothermal conversion efficiencies up to 70%. However, they are often expensive, synthetically complex, and can present issues of cytotoxicity and poor biodegradability, making them less suitable for sustainable or large‐scale applications. The second category comprises scalable, low‐cost materials more suitable for energy harvesting. For instance, graphene oxide and reduced graphene oxide coatings have demonstrated stable light‐to‐heat conversion and have been integrated into, e.g., TEGs.^[^ ^24^, ^25^ ^]^ Carbon nanotube (CNT) films have also been used in flexible photothermal systems due to their high thermal conductivity and absorption.^[^ ^26^, ^27^ ^]^ Lignin‐derived nanoparticles, sourced from industrial biomass waste, have shown photothermal efficiencies up to 22% under sunlight exposure and are being explored for sustainable photothermal use.^[^ ^28^ ^]^ Metal oxides such as CuO, Fe_3_O_4_, and TiO_2_ have also been used in composite coatings for light‐driven heating applications, though their performance is often limited by absorption capability.^[^ ^29^, ^30^, ^31^ ^]^ These systems generally offer better scalability and environmental compatibility than their biomedical counterparts, but often lack the fine tunability, biocompatibility, or biodegradability desirable for green energy solutions.

In the frame of the emerging bio‐based or bio‐inspired photothermal materials, Nature provides a compelling precedent. In coral tissues, FPs serve as light‐harvester in photosynthesis, UV‐filters, and photothermal buffers.^[^ ^32^ ^]^ Indeed, protein engineering allows tuning their absorption spectra and suppression of fluorescence, making FPs promising for light‐to‐heat conversion. Although FPs have been used in bioimaging,^[^ ^33^ ^]^ intracellular thermometry,^[^ ^34^ ^]^ and photothermal therapy,^[^ ^35^ ^]^ along with a few protein‐hybrid systems, such as protein–dye assemblies^[^ ^36^ ^]^ and albumin‐bound fluorophores,^[^ ^37^ ^]^ their use in energy harvesting devices remains unexplored yet. Besides FP production is is already industrially established, resulting in an overall low cost in large‐scale fermenter production.^[^ ^38^ ^]^

In this study, we present a proof‐of‐concept FP‐TEG device, in which the red‐emissive FP E2‐Crimson is embedded in a water‐processed polyethylene oxide (PEO) matrix and applied to a commercial thermoelectric module. E2‐Crimson, derived from DsRed‐Express2, offers broad absorption across the visible range (extinction coefficient, ε = 126000 M^−1^cm^−1^) and a low photoluminescence quantum yield (ϕ), ideal for heat generation.^[^ ^39^ ^]^ The photon‐to‐heat features of the FP‐polymer coatings were optimized in terms of *i*) the amount of E2‐Crimson, *ii*) the polymer matrix composition, and *iii*) the type of illumination (monochromatic and white LED) and its power. Indeed, the FP‐coatings can reach temperatures up to 60 °C under white LED illumination sources, achieving an overall electrical power of up to 232 ± 12 µW cm^−2^ that is highly sensitive to ON‐OFF cycles and appropriate for low‐power autonomous devices, such as digital thermometers, smoke detectors, and low‐power microcontrollers, among others.^[^ ^7^ ^]^ To our knowledge, this is the first report of FP‐based coatings applied to thermoelectric generators (TEGs), establishing the novelty of this approach. Overall, these findings herald FPs as a promising class of light‐to‐heat converters for emerging energy applications.

## 2 Results and Discussion

### 2.1 FP‐Polymer Coating Preparation and Characterization

**Figure 1:**
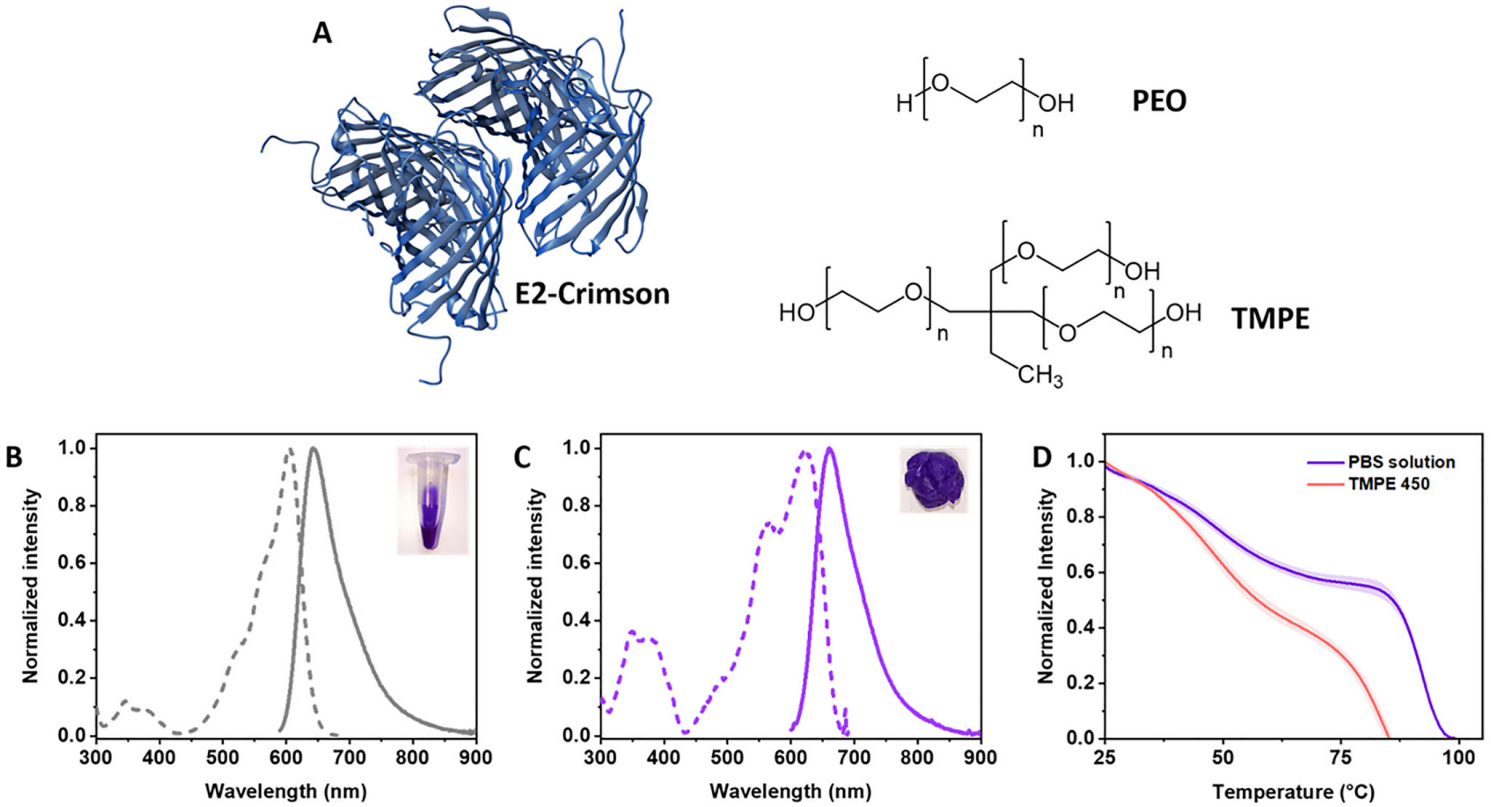
The structure of E2‐Crimson predicted using AlphaFold2^[^ ^42^ ^]^ and the structures of the polymer matrix components PEO and TMPE A). Excitation (dashed line; λ_em_= 700 nm) and emission (solid line; λ_exc_= 590 nm) spectra of E2‐Crimson in PBS buffer solution B) and in the TMPE‐450:PEO coating C). The insets show a picture of the protein solution and coating under ambient light illumination. Thermocycling measurement of E2‐Crimson in PBS buffer solution and TMPE‐450:PEO coating – see legend D).

A FP‐polymer coating was prepared by embedding E2‐Crimson into a polymer matrix composed of a blend of the branched trimethylolpropane ethoxylate (TMPE) stabilizer and the linear poly(ethylene oxide) (PEO) mixed in 4:1 mass ratio – **Figure** **1**A. Specifically, a PBS buffer aqueous solution of E2‐Crimson (39 nmol ‐ corresponding to the amount of fluorescent protein used during coating preparation; pH 7.4 PBS buffer; Figure 1B) was mixed with TMPE (M_n_ of 450) followed by the gradual addition of PEO (M_w_ of 5 × 10^6^ g mol^−1^) upon gentle stirring for 20 minutes. In molar terms, this corresponds to an approximate ratio of FP: TMPE: PEO = 1: 6800: 0.15, reflecting the large excess of TMPE relative to the protein, and the very small number of PEO chains due to its high molecular weight. They were then dried in a mold in a vacuum chamber at 3 mbar for 90 minutes, leading to a self‐standing coating – Figure 1C; see experimental section for more details.

**Figure 2:**
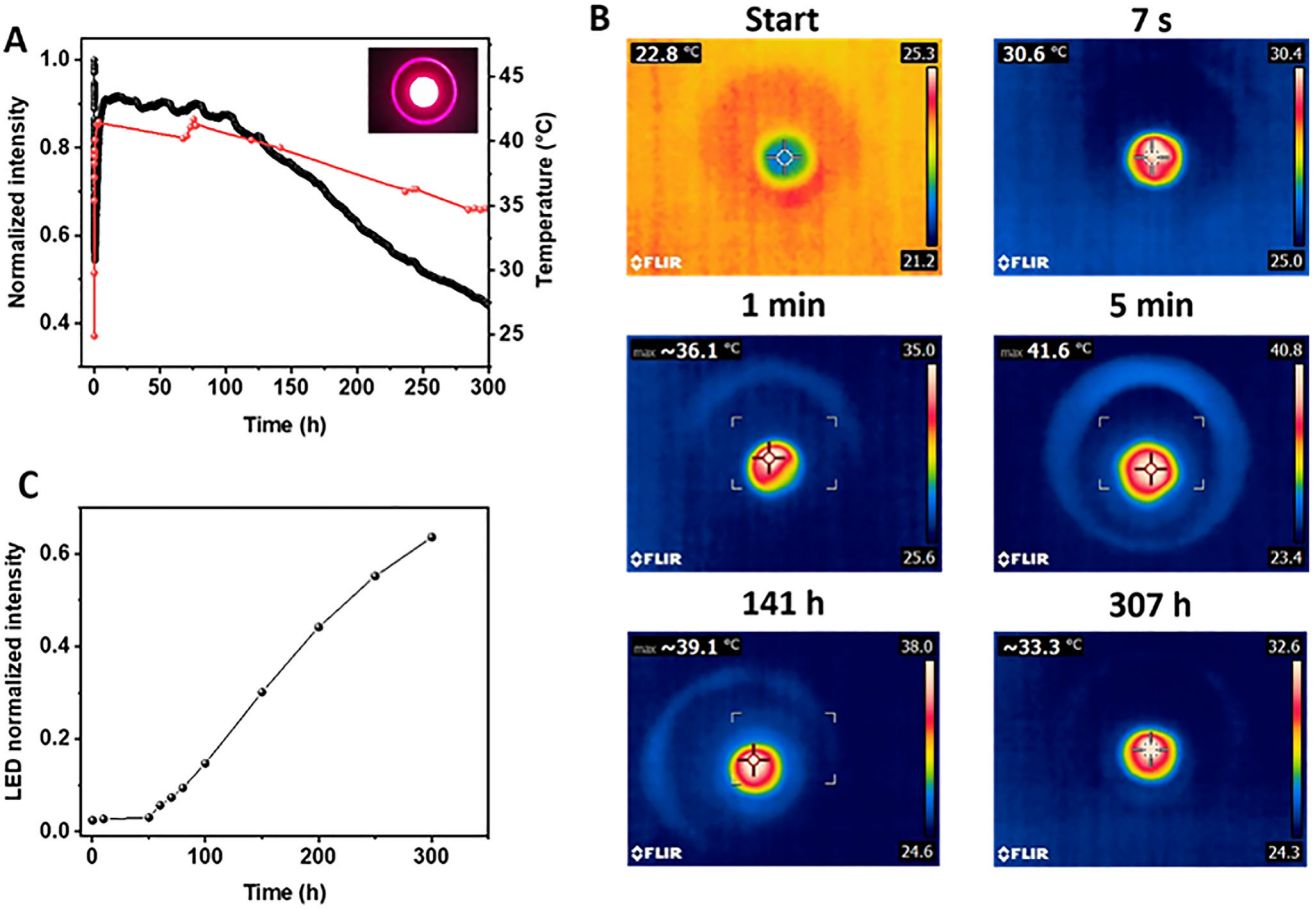
Temperature (red) and emission intensity (black) changes of E2‐Crimson coatings (39 nmol in TMPE‐450:PEO coating) at constant 590 nm incident irradiation of 40 ± 1 mW cm^−2^ A) and infrared images of the E2‐Crimson‐polymer coatings over time B). The inset shows a top‐view image of the working device. Emission intensity increase of the LED emission (590 nm) over time caused by the progressive deactivation of the protein‐polymer coating C).

These coatings feature a vivid violet color that closely matches that of the original protein solutions, as observed by the unaided eye – Figure 1B,C. This indicates that the FP did not experience a severe denaturation process upon forming the FP‐polymer coating. In general, the emission and excitation features of the coatings resemble those in PBS buffer solution – Figure 1. They show a similar emission band centered at *ca*. 660 nm associated to an excitation spectrum with two bands attributed to the protein chromophore in the ranges of 320–430 nm and 450–600 nm. In particular, both emission and excitation spectra bands are broadened in the polymer coatings and slightly red‐shifted compared to those in PBS buffer solution – Figure 1B,C and **Table** **1**. This is typically attributed to protein aggregation and alterations in the H‐bonding network at the β‐barrel structure due to the dense and water‐less polymeric network surrounding the protein upon drying, resulting in changes of the chromophore cavity.^[^ ^40^ ^]^ Indeed, these coatings show an increase in the average excited‐state lifetimes (*τ*) up to 1.20 ns and a reduction of the *ϕ* down to ca. 7% with respect to those in solution – Table 1. To further explore the impact of the protein aggregation on the photophysical features, the concentration of E2‐Crimson in the polymer coatings was varied from 19 nmol, to 39 nmol, and to 58 nmol − Figure S1 and Table S1 (Supporting Information). As the protein concentration increases, the emission band further red‐shifts accompanied by a small decrease in *𝜏* and *ϕ* values − Table S1 (Supporting Information). Finally, the thermodynamic stability of the proteins in the polymer coatings was assessed using modulated scanning fluorometry − Figure 1D and Figure S2 (Supporting Information), exhibiting a slight reduction of the refolding capability (*F*_r_) compared to that in PBS buffer solution − Table 1. This has been commonly observed for other red‐emitting FP‐polymer coatings, such as mCherry.^[^ ^41^ ^]^ Beyond steady‐state emission features, we also examined the photostability of the coatings and E2‐Crimson solution under continuous illumination. We executed a photostability analysis under continuous 590 nm LED illumination (40 mW·cm^−2^). After 100 hours, the E2‐Crimson coatings retained ca.88% of their initial emission, whereas purified protein solutions dropped down to ca. 36%, supporting a stabilizing effect of the polymer matrix – **Figure** **2**A; Figure S3 (Supporting Information).

**Table 1:** Spectroscopy and thermal figures‐of‐merit of E2‐Crimson in PBS buffer solution and TMPE:PEO coatings. The amount of E2‐Crimson in coatings was 39 nmol.

Matrix	*λ*_exc_ ^a^ ^)^ [nm]	*λ*_em_ ^b^ ^)^ [nm]	*FWHM* ^c^ ^)^ [nm]		*ϕ * ^d^ ^)^ [%]	*τ* ^e^ ^)^ [ns]	*k*_rad_ ^f^ ^)^ [s^−1^]	*k*_nrad_ ^g^ ^)^ [s^−1^]	*F_r_* ^h^ ^)^
PBS solution	606	641	73	12.5 ± 1.1		1.05	1.19	8.33	46.35
TMPE‐170:PEO	617	658	73	8.8 ± 0.4		1.40	0.63	6.51	33.38
TMPE‐450:PEO	616	660	77	6.8 ± 0.1		1.20	0.57	7.77	36.91
TMPE‐1014:PEO	620	656	76	8.1 ± 0.4		1.20	0.68	7.66	37.48

^a)^ Maximum excitation wavelength at *λ*_em_ = 700 nm;

^b)^ Maximum emission wavelength at *λ*_exc_ = 590 nm;

^c)^ Full width at half maximum of emission;

^d)^ Photoluminescence quantum yield at *λ*_exc_ = 590 nm;

^e)^ Average excited state lifetime at *λ*_exc_ = 375 nm and respective *λ*_em_;

^f)^ Radiative rate constant, ×10^−8^;

^g)^ Non‐radiative rate constant, ×10^−8^;

^h)^ Refolding capability.

To assess the photostability and photon‐to‐heat conversion efficiency of E2‐Crimson‐polymer coatings, they were first transferred onto an unmodified commercial LED with emission centered at 590 nm. The LED was operated to provide a 40 ± 1 mW cm^−^^2^ incident photon irradiance under ambient conditions (ca. 20 °C and 56% humidity), while the temperature and emission intensity changes of the protein‐polymer coatings were simultaneously monitored – see experimental section for details. As reference, the same configuration and operation conditions were applied with polymer coatings without E2‐Crimson. As shown in Figure 2A,B, E2‐Crimson coatings (39 nmol) experience a temperature rise up to 41 ± 2 °C upon continuous irradiation in the aforementioned conditions, while the reference coatings exhibit no significant temperature increase ≤ 5 ± 2^ o^C − Figure S4 (Supporting Information). The central‐to‐edge temperature gradient present in the coatings’ pictures in the Figure 2B reflects the higher light intensity at the center of the LED beam combined with greater heat dissipation at the coating edges, consistent with previously observed photothermal heating patterns in thin films.^[^ ^49^, ^50^ ^]^ Interestingly, the temperature rise quickly evolves within the first five minutes of operation – Figure 2B, reaching a plateau that holds over *ca*. 100 h followed by a slow linear decrease to reach a 10% loss of the temperature gradient at around 250 h – Figure 2A. The temperature decay should be related to the photo‐induced protein degradation via protonation, isomerization and/or oxidation of the protein chromophore,^[^ ^43^, ^44^, ^45^, ^46^, ^47^, ^48^ ^]^ as the emission intensity of the FP follows the same trend of the temperature (Figure 2A) and the LED emission intensity starts to linearly increase from 50 h on (i.e., less photon down‐converting efficiency) – Figure 2C. Indeed, over long working periods the FP‐polymer coatings became colorless as the chromophore is fully deactivated.

### 2.2 Optimizing the FP‐Polymer Coating

**Figure 3:**
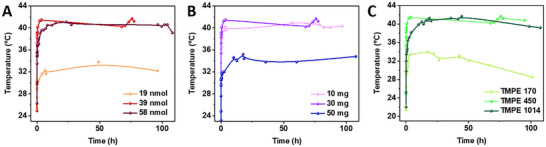
Temperature changes of E2‐Crimson TMPE:PEO coatings at constant 590 nm incident irradiation of 40 ± 1 mW cm^−2^ upon changing the amount of E2‐Crimson A), the amount of PEO B), and the type of TMPE C) – see legends for details.

Based on the above findings, we can conclude that the rise in the temperature of the coatings upon excitation is related to the non‐radiative deactivation mechanism of E2‐Crimson coupled to an efficient heat transfer across the FP‐polymer interface and the polymer network, keeping a positive temperature gradient relative to the reference TMPE:PEO coatings for approximately 300 hours. Thus, the temperature behavior of the FP‐polymer coatings could be fine‐tuned with respect to the protein content and the polymer composition. On one hand, the decrease of the protein amount from 39 nmol to 19 nmol led to a reduction in the maximum temperature down to 34 ± 2 °C, while a further increase of up to 58 nmol did not exert any change in the maximum temperature (41 ± 3 °C) − **Figure** **3**A. This is reasonable as the LED‐to‐protein emission intensity conversion is over half for the low‐content E2‐Crimson coatings, while it is already 100% for those with 39 nmol – Figure S5 (Supporting Information). On the other hand, coatings with constant amount of E2‐Crimson (39 nmol) and TMPE‐450, but with increasing amounts of PEO going from 10 mg, to 30 mg, and to 50 mg were also prepared − Figure 3B. Here, the rise in the temperature showed no significant change in the range of 10 to 30 mg (*ca*. 40 ± 5 °C), while the highest PEO amount (50 mg) reduces the maximum temperature down to 35 ± 1 °C. To rationalize these findings, differential scanning calorimetry (DSC) characterization was carried out – Figure S6 (Supporting Information). DSC of pristine coatings feature a glass transition temperature (*T*_g_) at ‐60 °C due to the presence of free TMPE regions and a melting process (*T*_m_) ranging from 30‐50 °C attributed to the crystalline PEO phase. The glass transition of PEO, which occurs around ‐53 °C in the pristine sample, is not discernible in the coating due to overlap with the prominent *T*_g_ of TMPE.

Furthermore, the increase of PEO leads to a large distribution of crystallite sizes, resulting in a broadening of the melting process with a *ca*. 38% increase of the enthalpy of the process − 20.72 J g^−1^ (50 mg) versus 15.04 J g^−1^ (30 mg), while the thermal features of the free TMPE gradually disappeared. The addition of E2‐Crimson (39 nmol) in this polymer series results in *i*) the earlier disappearance of the glass transition process in coatings with 30 mg PEO on, as the protein well‐intermixes with TMPE, and *ii*) a higher degree of broadening of the melting process with a strong reduction of the *T*_m_ down to 38 ± 1 °C for coatings with 50 mg PEO (56 ± 1 °C and 42 ± 1 °C for coatings with 10 and 30 mg PEO content) associated to a gradual increase of the enthalpy of the process –, i.e., 12.73 J g^−1^ (10 mg), 15.04 J g^−1^ (30 mg), and 20.72 J g^−1^ (50 mg). Thus, the low temperature rise for 50 mg PEO content could be related to the low melting temperature and high enthalpy expected from a higher PEO crystallite size distribution.

In addition to these thermal and photophysical considerations, we also assessed the environmental stability of the coatings. Given the water‐soluble and hygroscopic nature of PEO, coatings were evaluated for humidity resistance. Samples stored at ca. 56% relative humidity for 5 days lost ca. 14% of their original mass as they dried over time – Figure S7 (Supporting Information). Finally, we investigate the impact of the TMPE type, implementing stabilizers of M_n_ of 170, 450 and 1014 in a 4:1 mass ratio with PEO (30 mg) as well as a constant amount of 39 nmol E2‐Crimson – vide supra. In short, the photophysical and thermal features of the coatings are similar to those above described regardless of the type of stabilizers – Table 1 and Figure S8 (Supporting Information). However, the photon‐to‐heat conversion is dramatically impacted with an increasing maximum temperature values going from 34 ± 1 °C (TMPE‐170), to 41 ± 2 °C (TMPE‐450), and to 43 ± 1 °C (TMPE‐1014) – Figure 3C. This is attributed to the shift to higher melting temperatures of the polymer coatings upon increasing the molecular weight of the stabilizer – Figure S6 (Supporting Information). In addition, the coatings with TMPE‐1014 showed a slightly enhanced non‐reversible protein folding temperatures *F*_r_ of 37.48 – Table 1 and Figure S2 (Supporting Information). Hence, we identified the best protein‐polymer blend with 30 mg PEO and TMPE‐1014 (4:1 mass ratio) doped with 39 nmol of E2‐Crimson as the optimal composition to be implemented in TEGs.

### 2.3 Photon‐to‐Heat Conversion with FP‐Polymers

**Figure 4:**
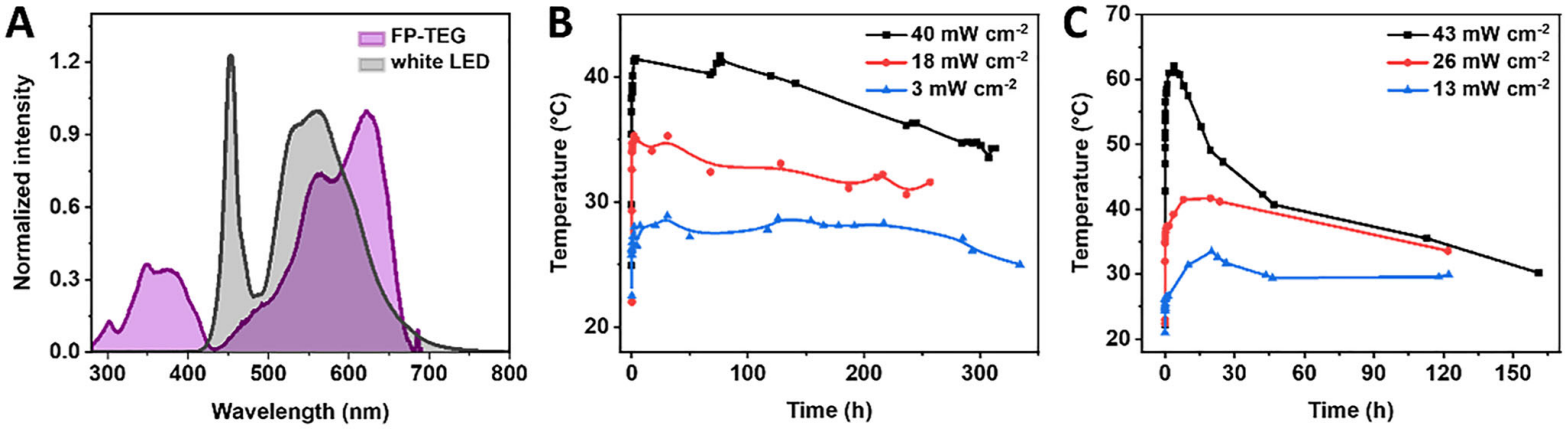
Overlapping of the normalized absorption spectra of E2‐Crimson protein‐polymer coatings with the emission spectra of the white LED A). Temperature changes of E2‐Crimson TMPE‐1014:PEO coatings at varying 590 nm B) and day white LED incident irradiation C).

At first, we evaluated the stability and photon‐to‐heat conversion efficiency of FP‐polymer coatings under different incident photon irradiances, that is, monochromatic 590 nm LED and white LED at different photon‐incident power densities – **Figure** **4**; see experimental section for details. In detail, the reduction of the 590 nm incident irradiation from 40 ± 1 mW cm^−2^ down to 18 ± 2 mW cm^−2^, and to 3 ± 1 mW cm^−2^ resulted in a reduction of the maximum temperature (41 ± 2 °C, 35 ± 2 °C, and 30 ± 1 °C, respectively) – Figure 4B. Likewise, white LED incident irradiation of 43 ± 2 mW cm^−2^, 26 ± 1 mW cm^−2^, and 13 ± 1 mW cm^−2^ resulted in a maximum temperature of 62 ± 3 °C, 42 ± 1 °C, and 34 ± 2 °C, respectively – Figure 4C. As noted above, reference coatings without E2‐crimson show no significant temperature increase also upon white LED illumination – Figure S4B (Supporting Information). Since surface temperature rise of the coating is proportional to the bottom incident radiant energy density (Figure S9, Supporting Information), the protein could function as a spectral filter, absorbing and dissipating energy near the surface, thereby reducing heat accumulation across the coating thickness. This might explain the reduction in the maximum photo‐induced coating temperature under both narrow‐band and white‐light irradiation.^[^ ^51^, ^52^ ^]^ No clear melting of the coating was observed. Instead, a gradual drying of the sample was noted (Figure S7, Supporting Information), which was further confirmed by an independent experiment in which the sample was kept at 60 °C for a couple of hours – Figure S12 (Supporting Information).

Given the measurable heating observed in E2‐Crimson loaded coatings, we next quantified their photothermal conversion efficiency under both narrow‐band and broadband illumination. This allowed us to calculate a maximum photothermal conversion efficiency of 29% and 48% for 590 nm and white LED illumination, respectively − see experimental section for details. These values are among the best reported for bio‐based/‐inspired photothermal converting materials, particularly lignin‐based derivatives – Table S2 (Supporting Information). Although some of these studies have reported higher maximum temperatures, they were obtained under significantly higher photon power densities of ca. 100 mW cm^−^^2^ and primarily targeted for solar light applications.^[^ ^28^ ^]^ While the above efficiency values are reported for the first minutes of illumination, prolonged operation results in a gradual decrease in efficiency, in line with the chromophore degradation shown as discussed in the photostability section‐Figures 2 and S3 (Supporting Information).

**Figure 5:**
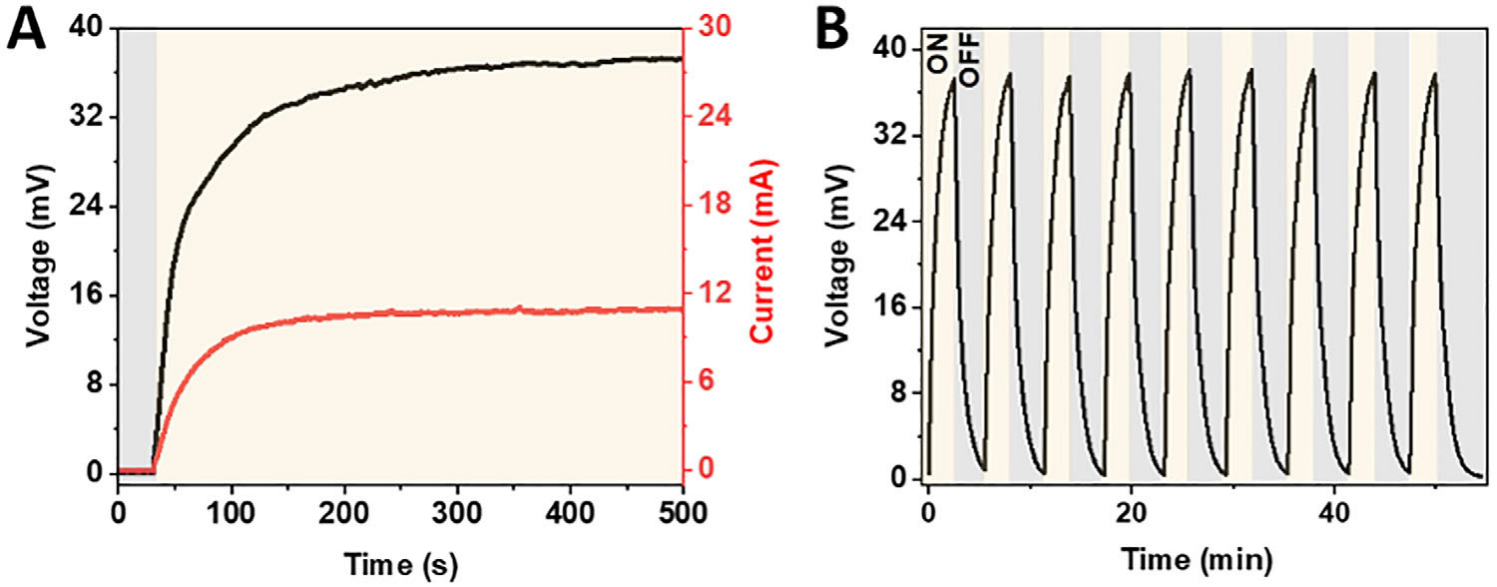
Voltage (black) and current (red) generation using FP‐TEG upon white LED irradiation of 43 ± 2 mW cm^−2^ A) as well as voltage generated in cycling ON‐OFF mode B). A video demonstration of the FP‐TEG charging a capacitor up to 10 mV under illumination, recorded without any external power boosters, is provided in the (Video S1, Supporting Information).

To further set in the relevance of proof‐of‐concept of a FP‐TEG, a commercial TEG module (TEC Microsystems) was coated with the above optimized protein‐polymer coating, while a copper heat sink was attached to the opposite side to enhance heat dissipation − Figure S12C (Supporting Information), experimental section for details. Upon illumination with the white LED source at incident photon density of 43 ± 2 mW cm^−^^2^, the thermal gradient generates an open‐circuit voltage of approximately 40 ± 3 mV cm^−^^2^ and a short‐circuit current of 10 ± 2 mA cm^−^^2^ − **Figure** **5**A. This results in a power of 232 ± 12 µW cm^−^^2^ − Figure S10 (Supporting Information) − that shows a high sensitivity to the light source with the voltage output rapidly decreasing when the illumination stops − Figure 5B. Reference devices with the TEG coated with FP‐free polymer confirm the key role of the FP excitation in the energy harvesting process – Figure S11 (Supporting Information). As a proof‐of‐concept demonstration, the generated electricity was used to charge a capacitor up to 10 mV, limited by the forward drop of the Schottky diode – Figure S14 (Supporting Information) and Video S1 (Supporting Information). While this voltage is insufficient to directly power conventional devices, the measured power output falls within the operational range of duty‐cycled IoT nodes, microcontrollers, and low‐power sensors when combined with appropriate power management circuitry, highlighting the potential of FP‐TEG for sustainable energy harvesting applications.^[^ ^7^ ^]^ Both aspects highlight the system's controllability with the incident light and future applicability.

Beyond performance metrics, the mechanical stability of the FP‐TEG system under operational stress is equally critical. Thermal cycling experiments revealed distinct behaviors at both the coating and device levels. SEM images of the FP coatings on glass substrates showed no observable morphological changes after up to 20 cycles, suggesting short‐term mechanical robustness of the coating itself – Figure S13 (Supporting Information). However, when applied to commercial TEG modules, partial delamination was observed after repeated cycling, indicating that poor adhesion to the device substrate remains a limitation – Figure S12C (Supporting Information). This underscores the need for further optimization of both the polymer matrix and the coating–substrate interface to improve mechanical durability under long‐term operational conditions.

The thermal contact quality at the coating–TEG interface is a key factor in determining overall performance, alongside mechanical integrity. To evaluate this, we estimated the thermal interfacial resistance using an energy balance approach that incorporated the absorbed light flux and the measured steady‐state temperature gradients, as described in the supporting information. The resulting interfacial resistance was 0.08 m^2^·K·W^−1^, even after accounting for the bulk thermal resistance of the coating. For comparison, strongly bonded polymer–ceramic interfaces typically exhibit resistances in the 10^−6^–10^−8^ m^2^·K·W^−1^ range, reflecting efficient heat transfer under pressure‐assisted or chemically bonded conditions.^[^ ^53^, ^54^ ^]^ In contrast, unbonded or lightly pressed interfaces are dominated by surface roughness and interfacial air gaps, which raise resistances to the 10^−3^–10^−1^ m^2^·K·W^−1^ range.^[^ ^55^, ^56^ ^]^ Our measured value thus falls within this unbonded regime, consistent with the absence of applied pressure or chemical bonding in the present configuration. These findings underscore the limitations of the current system and the need for interface engineering strategies, including applied pressure, thermal interface materials, or surface modification, to reduce thermal contact resistance.

## 3 Conclusion

This work successfully developed and characterized the first photon‐to‐heat energy harvesting fluorescent protein‐polymer coating that coupled to a commercial TEG shows a power gain of 232 µW cm^−2^ that is highly sensitive to the ON‐OFF cycles. What is more, photon‐to‐heat conversion efficiency is well placed at the state‐of‐the‐art of bio‐based/‐inspired photothermal materials – Table S2 (Supporting Information). This has been rationalized by using a high‐absorbing and low‐emissive E2‐Crimson protein and optimizing the thermal features of the coatings in terms of both, protein and polymer blend composition, as well as the type and intensity of the irradiation conditions. Looking ahead, this proof‐of‐concept FP‐TEG coatings hold significant promise for sustainable energy harvesting applications for the Internet of Things, indoor uses, and low‐power autonomous devices. However, future research should focus on enhancing thermal management capability, fine tuning protein‐polymer and coating‐TEG interfaces improving both thermal and mechanical coupling at the device interface to enhance long‐term performance and reliability, and increasing photo‐ and thermal‐stabilities of the FPs by engineering protein chromophore cavity and protein scaffolds and/or looking at other thermal stable proteins, such as phycobiliproteins. This could allow to operate at temperature closer to 100 °C that are required to enhance device performance. Overall, this work settles FP‐based photothermal materials as a promise avenue in sustainable and efficient energy‐harvesting.

## 4 Experimental Section

### E2‐Crimson Production

E2‐Crimson was produced in *E. coli* BL21 DE3 containing the plasmid pSW002. For cultivation the standard lysogeny broth medium (pH 7.4) was used. The cultivation was performed at 30 °C and 200 rpm for 3 days. After the cultivation the cells were washed and pelleted by centrifugation (4000 g, 20 min, 4 °C), before freezing −20 °C. The cell opening was performed by sonication using an amplitude of 80% and a 1 s pulse time paired with a 3 s pause for a process time of 8 min. The purification was done via size exclusion. After purification, the protein was flash‐frozen in PBS buffer with a 5 mg mL^−1^ concentration and stored at ‐80 °C. Before use, the proteins were thawed and centrifuged to remove aggregated proteins.

The E2‐Crimson amino acid sequence was: MNSTENVIKPFMRFKVHMEGSVNGHEFEIEGVGEGKPYEGTQTAKLQVTKGGPLPFAWDILSPQFFYGSKAYIKHPADIPDYLKQSFPEGFKWERVMNFEDGGVVTVTQDSSLQDGTLIYHVKFIGVNFPSDGPVMQKKTLGWEPSTERNYPRDGVLKGENHMALKLKGGGHYLCEFKSIYMAKKPVKLPGYHYVDYKLDITSHNEDYTVVEQYERAEARHHLFQ*

### Experimental Characterization Techniques

The photophysical studies were carried out using a FS5 Spectrofluorometer (Edinburgh Instruments) with the SC‐05/SC‐10 module for liquid/solid samples and the time‐correlated single photo‐counting module to determine τ. The data was then adjusted to a bi‐exponential decay fit using Origin 2019 (OriginLab Corporation, Northampton, MA, USA). To calculate the average lifetime for each FP‐coating, the following equation was used $<\tau {>}_{0}=\frac{\int_{0}^{x}t\;\sum a_{i}\;\exp\left(-\frac{t}{\tau_{i}}\right)\mathrm{dt}}{\int_{0}^{x}\sum a_{i}\;\exp\;\left(-\frac{t}{\tau_{i}}\right)\mathrm{dt}}=\frac{\sum a_{i}\tau_{i}^{2}}{\sum a_{i}\tau_{i}}$;^[^ ^57^ ^]^ where a_i_ was the amplitude fractions and τ_i_ were the lifetimes. The measurements were performed at room temperature. The photoluminescence quantum yields were measured using a Quantaurus‐QY Absolute PL quantum yield spectrometer (Hamamatsu Photonics). Radiative and non‐radiative rate constants were calculated using the following equations: $k_{rad}=\frac{\Phi }{\tau }$ and $k_{nrad}=\frac{1-\Phi }{\tau }$ where τ represents the average excited‐state lifetime and Φ photoluminescence quantum yield.

Modulated scanning fluorimetry (MSF) was performed as described in Svilenov et al.^[^ ^58^ ^]^ The Thermocycler CFX96 Touch Real_time PCR System (Bio‐Rad) was employed to perform MSF measurements. One standard program composed of heating and cooling cycles ranging from 25 °C to 99 °C was used to measure the progressive loss of florescence and the irreversible unfolding of the FPs studied in this work. The samples were heated 5 °C s^−1^ and held for 1 min at the temperature peak, followed by a recovery period of 5 min at 25 °C. The used concentration in this study was 7.5 µM of FPs either in a 50 µL volume of solution or in 10 mg of coating, were added per well to avoid saturation. The thermograms were buffer‐subtracted and normalized by the highest fluorescence readout of each sample. The data analysis was performed using Origin 2019 (OriginLab Corporation, Northampton, MA, USA). Mean values and standard deviations of quintuplicate were calculated and plotted. Non‐reversibility curves were obtained plotting the fluorescence values obtained at 25 °C. *F*_r_ (folding reversibility) was defined as the total area of the normalized intensity decay curve.

### Photothermal Conversion Efficiency

Calculation of the photothermal conversion efficiency was performed using the following equation: ^[^ ^28^, ^59^ ^]^

(1) $$\eta =\frac{Q}{E}=\frac{C_{p}\cdot m\cdot \Delta T}{P\cdot S\cdot t}$$

where Q and E correspond to the total heat generated and energy output of the LED, respectively. C_p_, m, and ΔT were specific heat capacity (for TMPE‐1014:PEO was 1.95 ± 0.31 J g^−1^ °C^−1^), mass of the coating (for the 590 nm LED irradiation experiment 196 mg, for the white LED irradiation 211 mg), change of the temperature within the first 20 minutes of irradiation (with the 590 nm LED irradiation: 16 ± 2 °C, for the white LED irradiation: 28 ± 1 °C). The total energy output (E) of the light source was calculated using irradiation of the LED (P; 40 ± 1 mW cm^−2^ and 43 ± 2 mW cm^−2^ for 590 nm and white LED irradiation), the irradiation area (S; 44 mm^2^), and time to reach given temperature gradient (t = 20 min). The specific heat capacities were quantified as indicated the next section.

### Preparation and Characterization of the FP‐Polymer Coatings

All reagents were purchased from Sigma‐Aldrich, specifically: 1,1,1‐Tris‐(hydroxymethyl)‐propan‐ethoxylat (TMPE; M_n_ of ≈ 170, 450, 1014) and linear polyethylene oxide (PEO; M_w_ of ≈ 5 × 10^6^ g mol^−1^). A solution of the protein was added to TMPE and stirred together; next PEO was added to the mix. The sample was dried for 90 minutes in a vacuum chamber at 3 mbar. Differential scanning calorimetry (DSC) was conducted using a Q200 DSC instrument from TA Instruments, with a heating rate of 20 °C min^−1^. Specific heat capacity was determined by differential scanning calorimetry using standard sapphire test method (ASTM E1269).^[^ ^60^ ^]^ The obtained value was an average of five independent measurements. *Scanning electron microscopy* (SEM) images were recorded from a DSM 940A (Zeiss, Germany). The sample holders were mounted by sticking a double‐sided adhesive tape on an aluminum stub at the bottom, and a glass disk plate on the top. The samples were directly sticked to the disk plate prior to gold‐palladium mixture sputtering. SEM images were obtained using 20 keV electron energy, using the secondary electron detector for images collection.

### Photostability and Temperature Characterization

The setup was created using an unmodified commercial 590 nm LED (WINGER WEPYE1‐S1 Power LED Star) and white LED (WINGER WEPWW1‐S1 Power LED Star white) as the pumping source. To perform photostability and photothermal conversion measurements, the protein‐polymer coatings were placed directly on top of an unmodified commercial LED driven at different currents to control the incident photon irradiation power at the surface of protein‐polymer coating. The emission spectra were measured using an Avantes Spectrometer 2048L (300 VA grating, 200 µm slit, CCD detector) connected to an AvaSphere 30‐Irrad Integrated sphere, with temperature monitored via a FLIR ETS320 thermographic camera. The employed power source was a Keithley 2231‐A‐30‐3. All the reported values were an average of three independent measurements and the error relates to standard deviation.

### FP Integration in Thermoelectric Generators

The TEG was purchased from TEC Microsystems and a copper heat sink was attached to its cold side. The hot side was covered with the protein coating. The sample was illuminated using commercial LEDs: 590 nm LED (WINGER WEPYE1‐S1 Power LED Star) or white LED (WINGER WEPWW1‐S1 Power LED Star). The open‐circuit potential and short‐circuit current generated by TEG were registered using a potentiostat PGSTAT204, Metrohm Autolab, by means of a chrono amperometry and a chrono potentiometry. The generated power was estimated using the current density‐voltage (J–V) characteristics.^[^ ^61^ ^]^ The reported value was an average of four independent measurements and the error relates to standard deviation.

### Estimation of Thermal Interfacial Resistance

To estimate the thermal interfacial resistance between the coating and the thermoelectric generator (TEG), an energy balance model based on steady‐state thermal measurements were employed and estimated convective and radiative losses to the ambient environment.^[^ ^62^ ^]^ This approach, while approximate, was suitable for capturing the relative contribution of the interface to overall heat transfer resistance in a proof‐of‐concept system. The total power absorbed by the coating was calculated using the measured white LED irradiance (*I*_in_ = 70 mW cm^−2^; a higher irradiance level was used in this study to ensure a measurable and reliable thermal gradient across the FP–TEG interface) and the experimentally determined absorptance of the coating (e.g.*, η*_abs_ = 0.786 at 470‐650 nm regime). This gave an absorbed heat flux of 0.04674 W over the illuminated area (A = 8.5 ·10^−5^ m^2^; Q_in_ = *η*_abs_· *I*_in_·A). Heat losses from the coating surface to the ambient environment were estimated using standard convective and radiative heat transfer equations −, i.e., Q_conv_ = h ·A (T_coating_‐T_amb_); Q_Rad_ = ε · σ · A (T_coating_^4^‐T_amb_^4^). Here, a convective heat transfer coefficient of 5.0 W·(m^2^·K)^−1^ − h = (m · C_p_) / (A · τ) − and an emissivity (ε; FLIR Thermal Camera using black tape as reference) of 0.85 were estimated, resulting in convective (Q_conv_) and radiative losses (Q_Rad_) of 0.01615 W and 0.01898 W, respectively. Subtracting these losses from the absorbed heat provided an estimate of the net heat flux conducted through the coating into the TEG (Q_TEG_ = 0.01161 W). The temperature drop across the coating–TEG interface was taken from surface temperature measurements: 333.15 K at the coating surface and 305.15 K at the TEG interface. Using this gradient, the total thermal resistance from the coating surface to the TEG was calculated as: R_total_ = ΔT · Q_TEG_^−1^ = 2411.8 K·W^−1^. This total includes both the bulk thermal resistance of the coating and the interfacial resistance at the TEG contact. The bulk resistance was calculated from the measured coating thickness (d, 1.5 mm) and an estimated thermal conductivity (k) of 0.012 W(m·K)^−1^ (relative value to reference Teflon under time‐controlled heating process), giving: R_bulk_ = d · (k·A)^−1^ = 1470.6 K·W^−1^. Subtracting the bulk contribution, the remaining interfacial resistance was: R_interface_ = R_total_‐R_bulk_ = 941.2 K·W^−1^. This corresponds to an area‐normalized interfacial thermal resistance of 0.08 m^2^·K·W^−1^. This estimation assumes uniform heat flow and contact area, and does not account for effects, such as micro‐scale delamination, porosity, or lateral heat spreading. The interfacial resistance reported here should therefore be interpreted as a first‐order approximation. More precise measurements using direct methods were planned for future work.

## Conflict of Interest

The authors declare no conflict of interest.

## Supporting information



Supporting Information

Supporting VideoS1

## Data Availability

The data that support the findings of this study are available from the corresponding author upon reasonable request.
