# Supplementary material for: Photon‐to‐Heat Energy Harvesting Fluorescent Protein Coatings for Thermoelectrics
Source: Adv Sci (Weinh). 2025 Dec 8;13(8):e22088. doi: 10.1002/advs.202522088 (PMC12884722; doi:10.1002/advs.202522088)
Supplement: Supplementary file 1 — Supporting Information [file ADVS-13-e22088-s001.docx]

Supporting Information

**Photon-to-Heat Energy Harvesting Fluorescent Protein Coatings for Thermoelectrics**

***Anna Zieleniewska,* Sriram Kunchapudi, Stephanie Willeit, and Rubén D. Costa****

**Technical University of Munich, Campus Straubing for Biotechnology and Sustainability, Chair of Biogenic Functional Materials, Schulgasse, 22, Straubing 94315, Germany.**

**E-mail:** ruben.costa@tum.de**; anna.zieleniewska@tum.de**


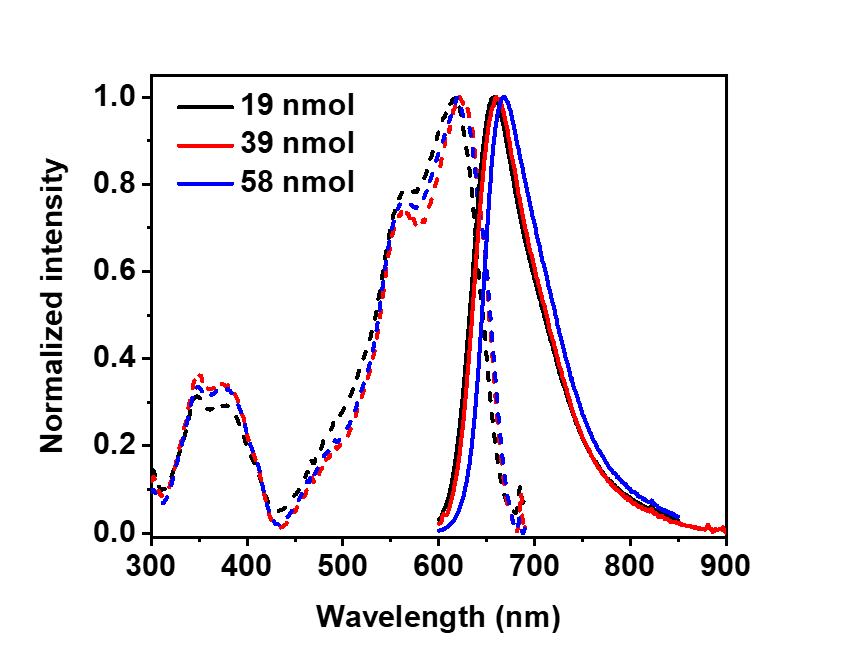


**Figure S1.** Excitation (dashed line; λ_em_= 700 nm) and emission (solid line; λ_exc_= 590 nm) spectra of polymer coatings bearing different amounts of E2-Crimson (see legend).

**Table S1.** Photophysical properties of FP-based coatings with varying amount of E2-Crimson.

| Protein | Amount of  E2-Crimson  (nmol) | Photophysical characterization | | | | | | |
| --- | --- | --- | --- | --- | --- | --- | --- | --- |
|  |  | *λ*_exc_ ^a)^  [nm] | *λ*_em_^b)^  [nm] | *FWHM*^c)^  [nm] | *φ*^d)^  [%] | *τ* ^e)^  [ns] | *k*_rad_^f)^  [s^-1^] | *k*_nrad_^g)^  [s^-1^] |
| E2-Crimson | 19 | 617 | 658 | 77 | 7.0 ± 1.3 | 1.25 | 0.56 | 7.44 |
|  | 39 | 622 | 660 | 77 | 6.8 ± 0.11 | 1.20 | 0.57 | 7.77 |
|  | 58 | 620 | 669 | 76 | 6.0 ± 0.9 | 1.20 | 0.50 | 7.83 |

*^a)^* Maximum excitation wavelength at *λ*_em_ = 700 nm; *^b)^* Maximum emission wavelength at *λ*_exc_ = 590 nm; *^c)^* Full width at half maximum of emission;*^d)^* Photoluminescence quantum yield at *λ*_exc_ = 590 nm; *^e)^* Average excited-state lifetime at *λ*_exc_ = 375 nm and respective *λ*_em_; *^f)^* Radiative rate constant, ×10^−8^; *^g)^* Non-radiative rate constant,×10^−8^.


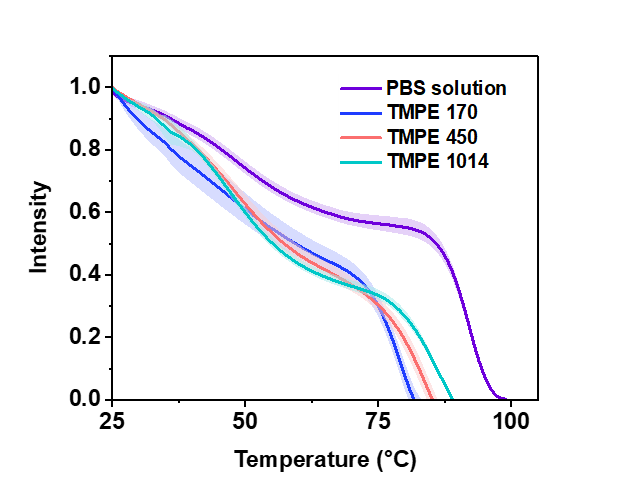


**Figure S2.** Modulated scanning fluorimetry experiments of E2-Crimson in aqueous PBS buffer solution and in TMPE:PEO (4:1 w/w) coatings using TMPEs of different M_n_.


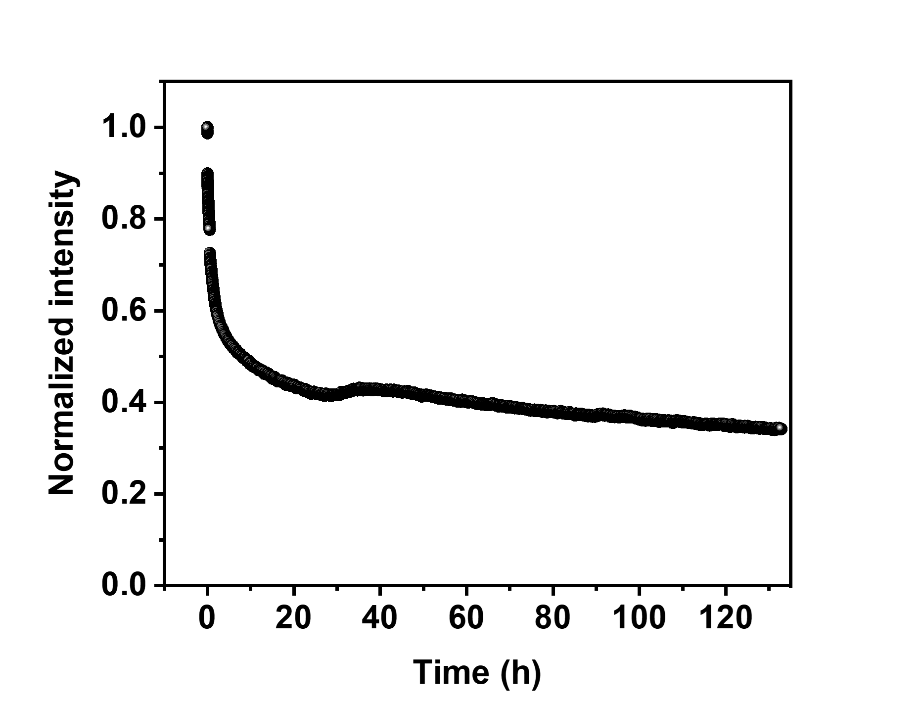


**Figure S3.** Emission intensity decay profile of E2-Crimson solution under 590 nm irradiation at 40 mW cm^-2^ in ambient conditions.


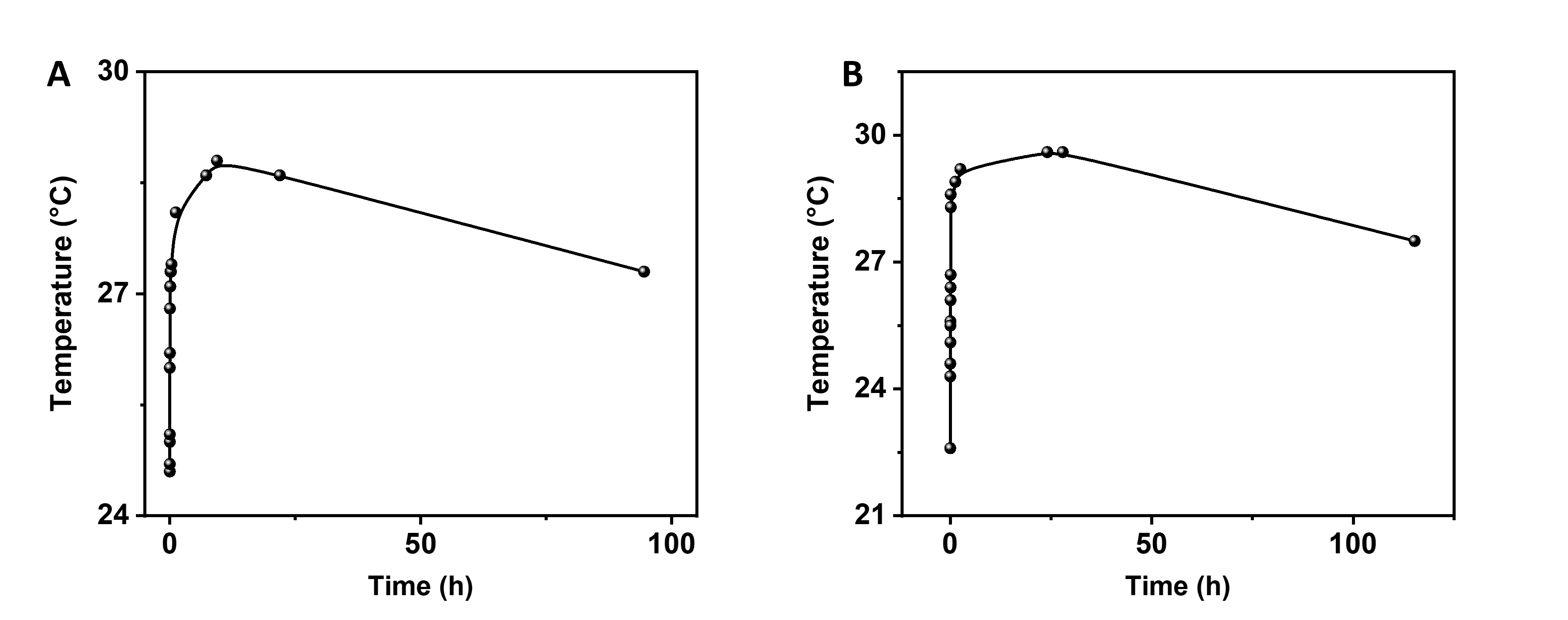


**Figure S4.** Temperature increase upon (A) 590 nm LED (40 ± 1 mW cm^-2^) and (B) white LED (43 ± 1 mW cm^-2^) illumination of reference TMPE-450:PEO coatings without protein.


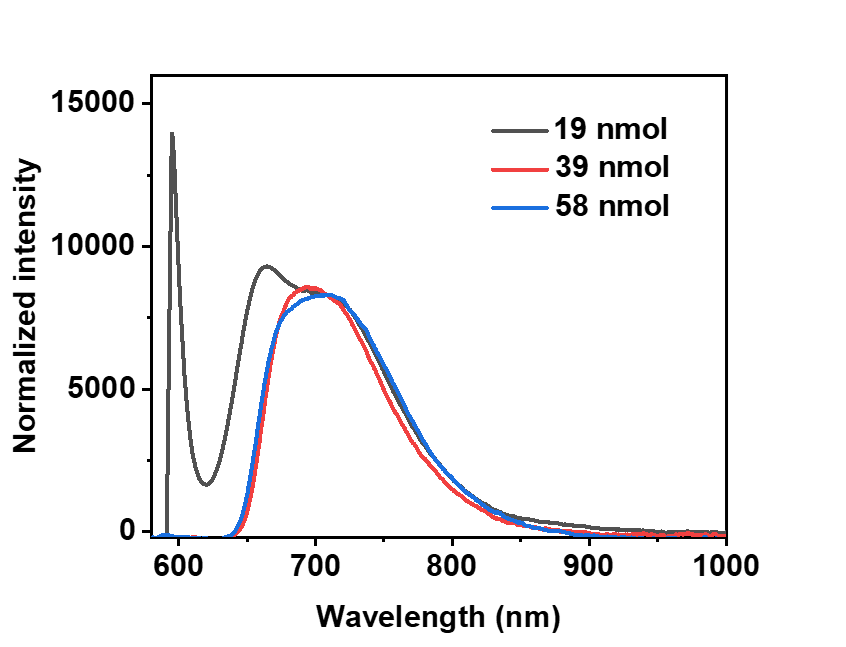


**Figure S5.** Initial fluorescence spectra of the device representing 590 nm LED-to-protein emission conversion with different amounts of E2-Crimson – see legend.


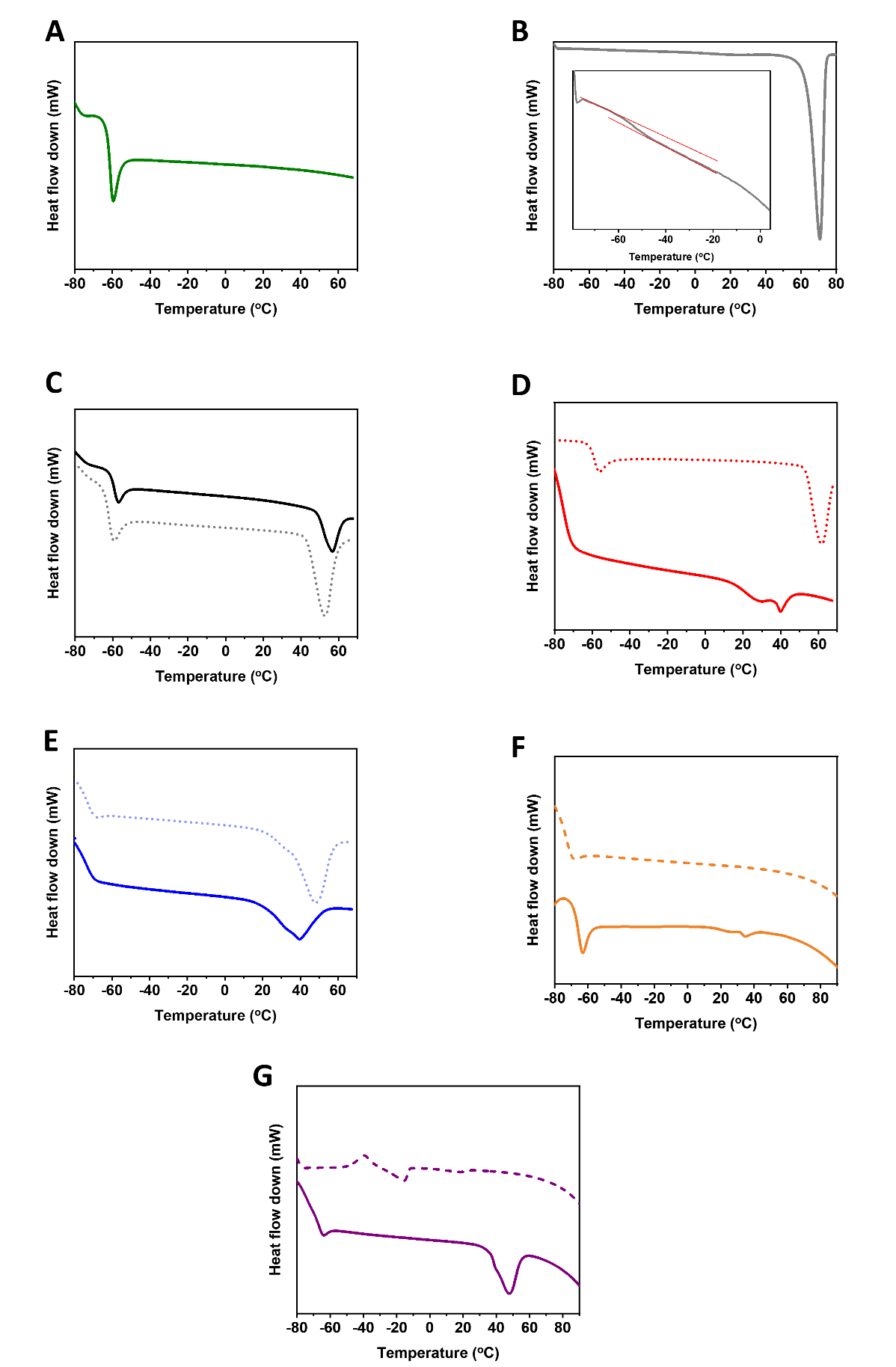


**Figure S6.** Differential scanning calorimetry (DSC) results comparing pristine TMPE (A), pristine PEO with the T_g_ temperature indicated in the insert (B), TMPE-450:PEO with increasing PEO mass from 10 mg (C), to 30 mg (D), and to 50 mg (E), as well as TMPE-170:PEO (F) and TMPE-1014:PEO coatings (G) of mass ratios 4:1 (30 mg of PEO) without protein (dashed lines) and with E2-Crimson (39 nmol; solid lines).


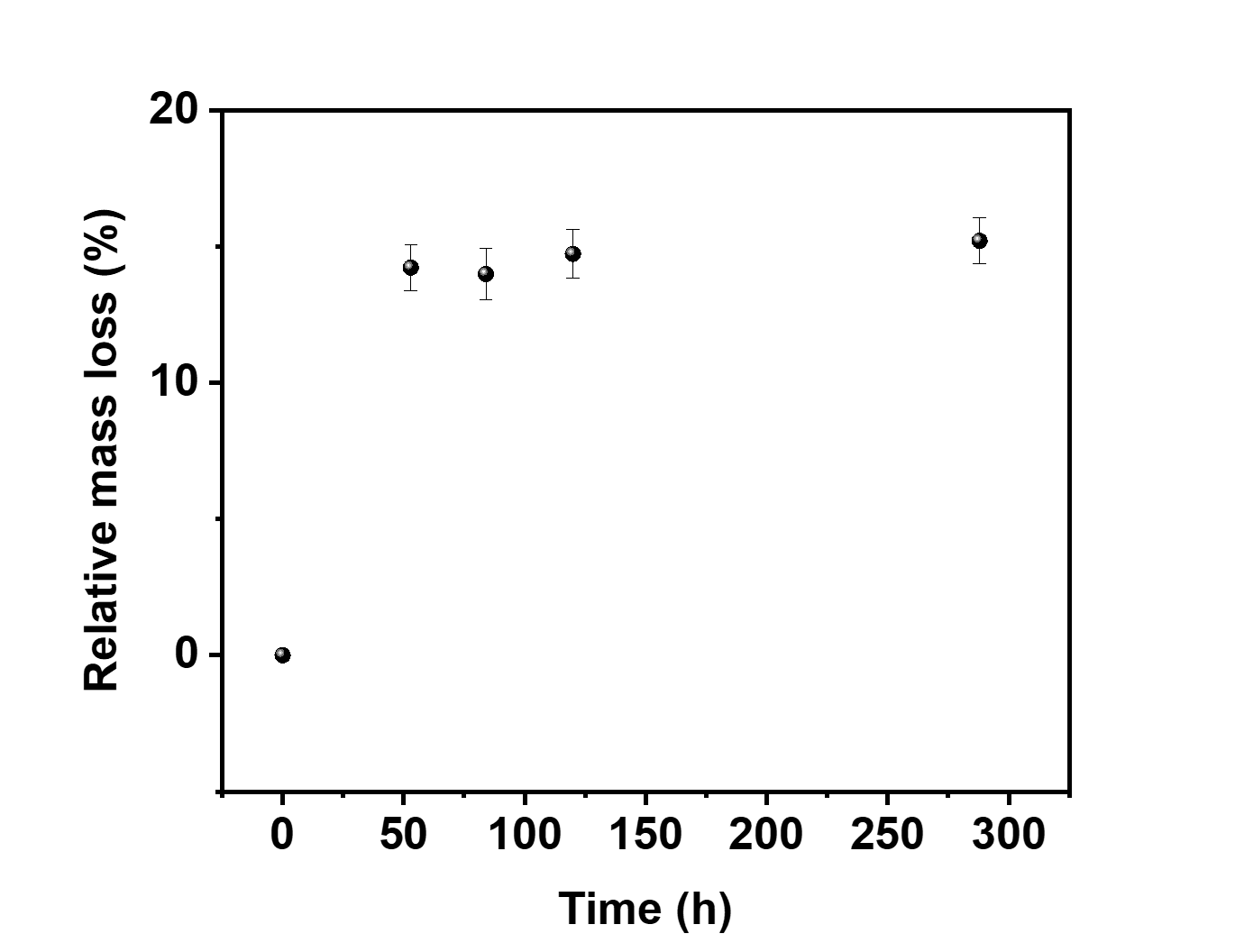


**Figure S7.** Relative mass loss of the sample stored in ambient conditions (56% RH and 20 °C).


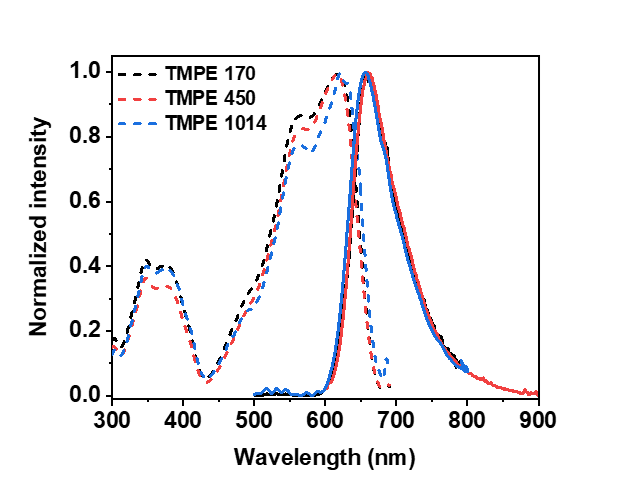


**Figure S8**. Excitation (dashed line; λ_em_= 700 nm) and emission (solid line; λ_exc_= 590 nm) spectra of polymer coatings bearing the same amounts of E2-Crimson (39 nmol), but different type of TMPE − see legend.

**Table S2.** Comparative analysis of photothermal conversion properties of bio-based/-inspired materials.

| **Material** | **Light source** | **Maximum temperature** | **Maximum photothermal conversion efficiency** | **Reference** |
| --- | --- | --- | --- | --- |
| Lignin nanoparticles in chitosan matrix | Solar simulator  100 mW cm^-2^ | 41 ^º^C | - | *ACS Appl. Mater. Interfaces* **2022**, *14*, 12693. |
| Lignin nanoparticles | Solar simulator  100 mW cm^-2^ | 63 ^º^C | 22% | *ACS Appl. Mater. Interfaces* **2021**, *13*, 7600 |
| Lignin nanoparticles in PVA matrix | Solar simulator  100 mW cm^-2^ | 55 ^º^C | - | *ACS Appl. Mater. Interfaces* **2021**, *13*, 7600. |
| This work | 590 nm LED  40 ± 1 mW cm^-2^ | 41 ^º^C | 29% |  |
| This work | White LED  43 ± 2 mW cm^-2^ | 62 ^º^C | 48% |  |


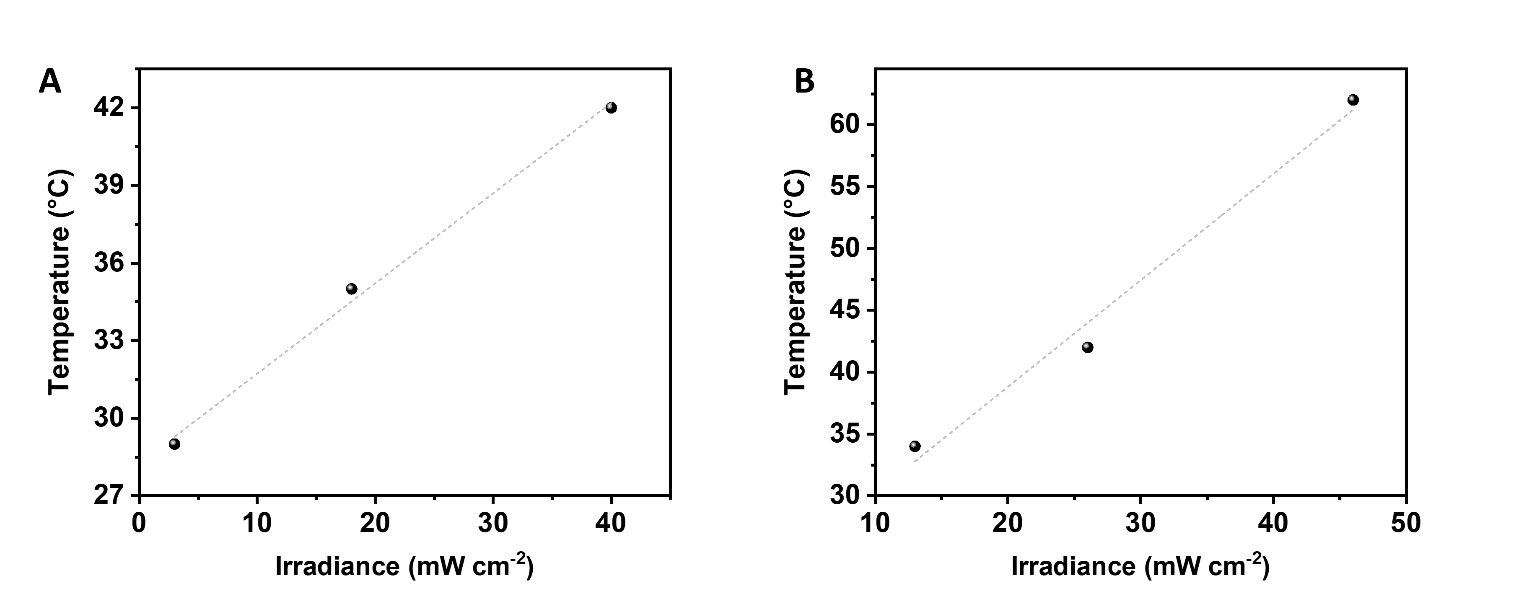


**Figure S9.** Relationship between irradiance and coating surface temperature under (A) 590 nm illumination and (B) white LED illumination.


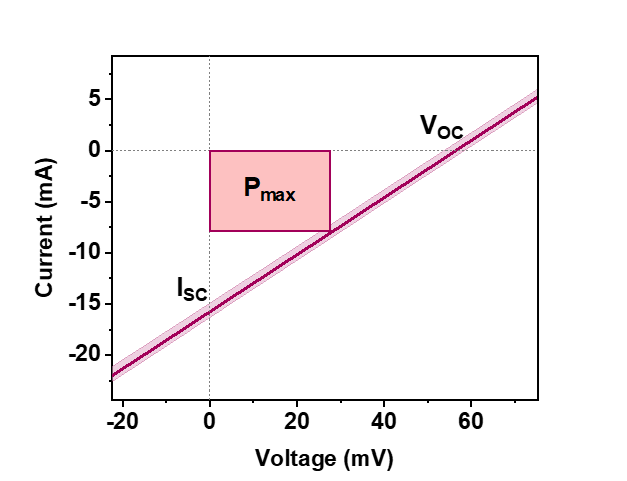


**Figure S10.** Current–voltage (I–V) characteristics of FP-TEG device under white LED illumination (43 ± 2 mW cm^-2^), V_OC_, I_SC_, and P_max_ correspond to the open-circuit voltage, short-circuit current, and maximum generated power, respectively.


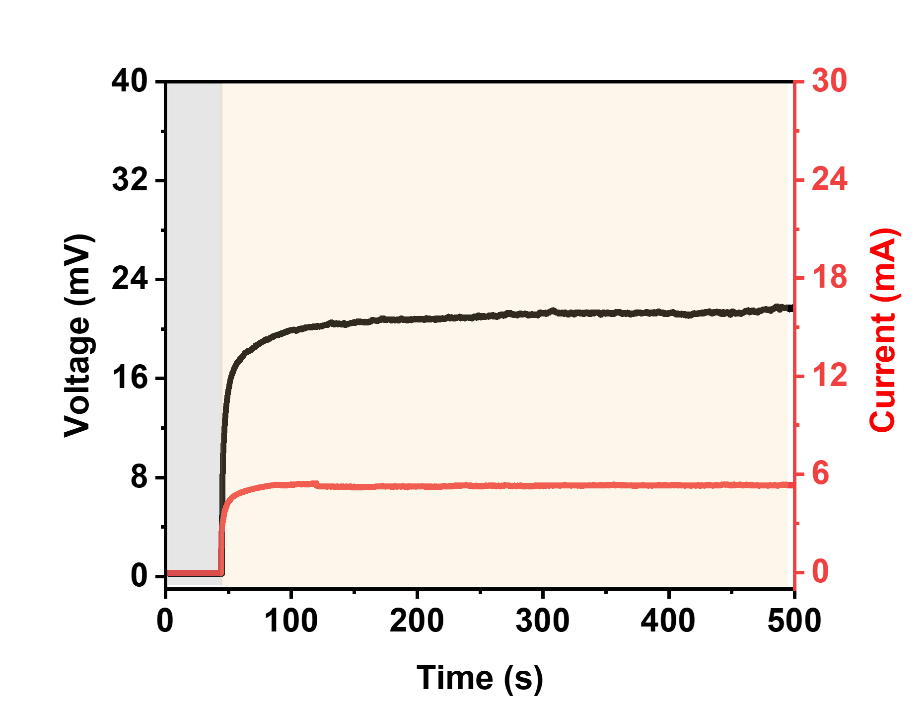


**Figure S11.** Voltage (black) and current (red) generated by the reference coating (polymer coating without E2-crimson) on TEG under white LED irradiation (43 ± 2 mW cm⁻²).


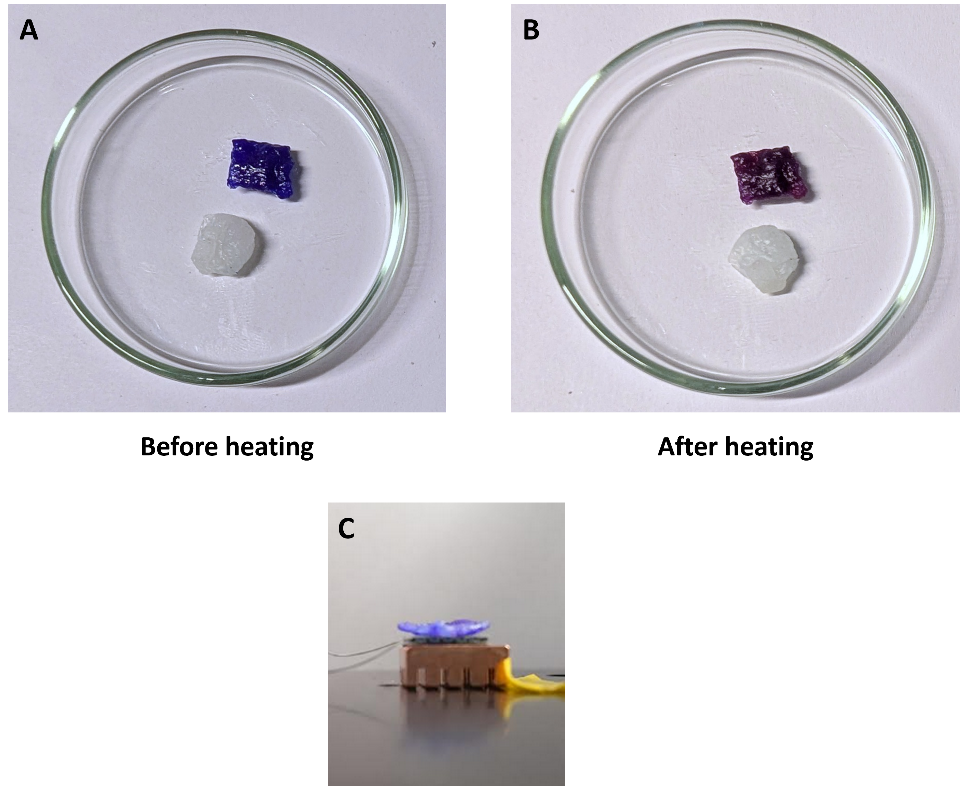


**Figure S12.** Pictures presenting E2-Crimson and reference coating before (A) and after (B) holding them at 60 ^o^C for 9 hours. After long-term experiments partial delamination of the coating from TEG surface was observed (C).


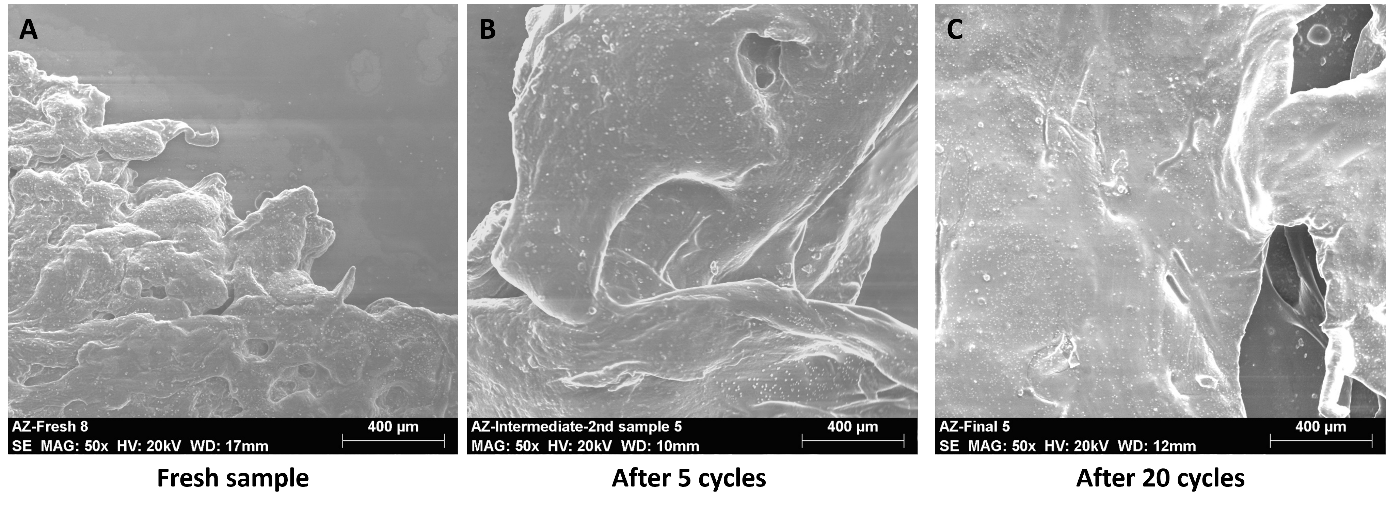


**Figure S13**. Scanning electron microscope (SEM) images of the FP–TEG coating surface (A) before and after white LED-induced photothermal cycling : (B) 5 and (C) 20 cycles.

**Experimental setup for capacitor charging demonstration**

To demonstrate the functional capability of the FP–TEG system under practical operating conditions, we performed a capacitor charging experiment using a white LED light source. The coated side of the TEG was illuminated using a white LED array positioned above the device. The opposite (cold) side of the TEG was contacted to the copper heat sink, allowing passive heat dissipation. The electrical output of the TEG was connected to an electrolytic capacitor through a Schottky diode to block reverse current and emulate a basic energy harvesting circuit. The capacitor was initially discharged, and its voltage rise was monitored using a digital multimeter to avoid influencing the charge behavior. No active power conditioning or voltage regulation was employed in this test. The experiment aimed to verify whether the thermal gradient induced by LED illumination alone could generate sufficient voltage and current to steadily accumulate energy in a conventional storage element.

The rise in capacitor voltage confirmed that the FP–TEG system can function as a self-powered energy harvester, even under modest and spectrally limited illumination conditions. While the total energy harvested was small, this proof-of-concept experiment supports the feasibility of integrating photothermal coatings into compact, low-power, and intermittently active electronic systems.


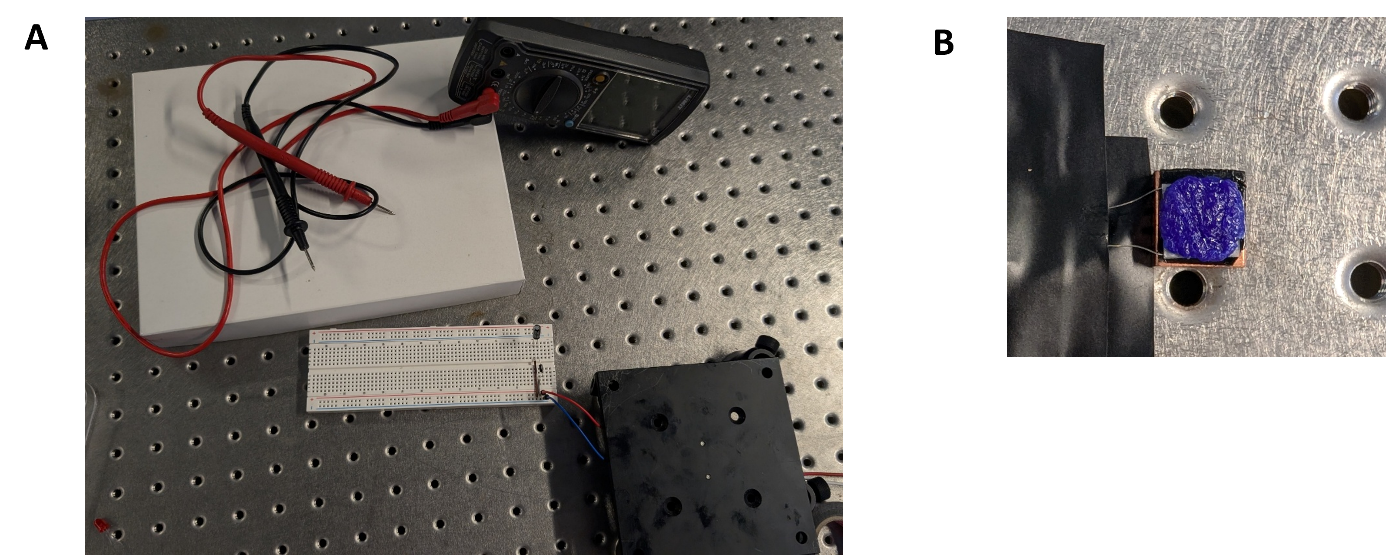


**Figure S14**. (A) Experimental setup used to demonstrate capacitor charging with the FP–TEG device. (B) Picture of FP coating on commercial TEG.
